# Research on the Construction of Grain Food Multi-Chain Blockchain Based on Zero-Knowledge Proof

**DOI:** 10.3390/foods12081600

**Published:** 2023-04-10

**Authors:** Boyang Zhang, Jiping Xu, Xiaoyi Wang, Zhiyao Zhao, Shichao Chen, Xin Zhang

**Affiliations:** 1 Beijing Key Laboratory of Big Data Technology for Food Safety, Beijing Technology and Business University, Beijing 100048, China; zbeyond_0712@163.com (B.Z.); xujiping@139.com (J.X.); zhaozy@btbu.edu.cn (Z.Z.); 2 Key Laboratory of Industrial Internet and Big Data, China National Light Industry, Beijing Technology and Business University, Beijing 100048, China; 3 Beijing Institute of Fashion Technology, Beijing 100105, China; wangxy@btbu.edu.cn; 4 State Key Laboratory for Management and Control of Complex Systems, Institute of Automation, Chinese Academy of Sciences, Beijing 100190, China; shichao.chen@ia.ac.cn

**Keywords:** blockchain, multi-chain, privacy encryption, zero-knowledge proof, consensus algorithm, food safety

## Abstract

As the main food source of the world’s population, grain quality safety is of great significance to the healthy development of human beings. The grain food supply chain is characterized by its long life cycle, numerous and complex business data, difficulty defining private information, and difficult managing and sharing. In order to strengthen the ability of information application processing and coordination of the grain food supply chain under many risk factors, an information management model suitable for the grain food supply chain is studied based on the blockchain multi-chain technology. First, the information on key links in the grain food supply chain is analyzed to obtain privacy data classifications. Second, a multi-chain network model of the grain food supply chain is constructed, and based on this model, the hierarchical encryption and storage mode of private data as well as the relay cross-chain communication mode, are designed. In addition, a complete consensus process, including CPBFT, ZKP, and KZKP algorithms, is designed for the global information collaborative consensus under the multi-chain architecture. Finally, the model is verified through performance simulation, theory analysis, and prototype system verification in terms of its correctness, security, scalability, and consensus efficiency. The results show that this research model effectively reduces the storage redundancy and deals with problems of data differential sharing in traditional single-chain research, as well as provides a secure data protection mechanism, a credible data interaction mechanism, and an efficient multi-chain collaborative consensus mechanism. By attempting to apply blockchain multi-chain technology to the grain food supply chain, this study provides new research ideas for the trusted protection of data and information collaborative consensus in this field.

## 1. Introduction

“Hunger breeds discontentment.” Human beings have been eating food crops for thousands of years. With the improvement in productivity, people’s diets have expanded from wheat to beans and rice. According to Statista, the global grain production is 2190 million tons in 2019/20, 2220 million tons in 2020/21, and 2753 million tons in 2021/22, increasing year by year. Food quality security problems are also becoming even more acute. Pesticide residues, pests, and diseases in crops during planting, heavy metal pollution in soil [1], heavy metals and illegal additives during storage and processing all have had a serious impact on food quality security [2]. In addition, since the outbreak of COVID-19, the global epidemic spread caused by food cold chains and supply chain logistics has occurred from time to time, which undoubtedly caused serious harm to people’s life and health as well as social stability [3]. Therefore, ensuring food quality safety and strengthening the information management of the whole grain chain is of great significance in safeguarding people’s quality of life and health and stability in the international community.

Traditional grain food quality security supervision is mostly centralized management models, and data are vulnerable to tampering and other attacks, which cannot effectively guarantee data security. It is also difficult to trace trusted data or achieve data sharing [4,5,6]. In order to solve the above problems, it has become a research hotspot in recent years to explore the application of new generation information technologies, such as blockchain and artificial intelligence, to agricultural products and food supply chain systems [7,8]. Blockchain is a decentralized, shared ledger that combines data blocks into a specific data structure in the form of a chain in chronological order and ensures that data are not forged or tampered with through cryptographic algorithms [9]. Some researchers have applied blockchain technology to agricultural products and food supply chains and have built a safe and efficient supply chain information traceability system through the traceability of blockchain combined with barcodes, RFID, trusted identification codes and other technologies [10,11,12,13]. Some researchers built an information supervision model for agricultural products and food supply chains with smart contract technology, aiming to strengthen the supervision of enterprise information [14,15,16]. The above studies have described information interconnection, data dynamic processing, and the information security and credibility of agricultural products and food supply chains, but these studies are all based on single-chain models. In an actual business scenario, the grain food supply chain is characterized by a long life cycle, numerous participation links, complex business data, and cross-regional supply chain entities. Although the traditional single-chain model breaks the information islands among enterprises and realizes the data interconnection, the consensus efficiency of the blockchain and the timeliness of data interaction are seriously affected by the huge and complicated data throughput of the grain supply chain. The joint maintenance of a ledger by the whole process nodes also causes a storage burden to the blockchain, and the information among enterprises cannot achieve differentiated sharing and management [17,18].

With the development of the multi-chain technology of blockchain, some researchers have also explored the application of multi-chain technology to agricultural products and food supply chain systems [19,20,21,22]. Multi-chain can effectively reduce the storage redundancy of traditional single-chain blockchain and isolate different business data, but it also leads to new challenges such as inter-chain cross-chain data communication and supply chain information coordination, etc. Researchers’ studies on multi-chain-based grain food supply chains are all from the perspectives of trusted supervision and trusted traceability of supply chain, ignoring the privacy rights division of different information of enterprises, governments and consumers, and the information coordination of the grain food supply chain from the perspective of multi-chain. A zero-knowledge proof is a cryptographic protocol that runs between the prover and the verifier, featuring completeness, soundness, and zero knowledge. The openness of blockchain brings serious privacy risks, while zero-knowledge proof can provide reliable privacy verification protection schemes [23]. A zero-knowledge proof can prove to the verifier the knowledge it acquires on the premise that the prover does not disclose any knowledge-related information and proves that data privacy is effectively guaranteed to provide a feasible scheme for complex information coordination in the grain food supply chain. Therefore, this paper builds a grain food multi-chain blockchain model based on a zero-knowledge proof, which integrates privacy encryption, digital signature, multi-chain and cross-chain technologies, etc. To realize the information hierarchical management of grain food supply chain and the trusted interaction of data among chains. Based on a zero-knowledge proof, a consensus mechanism in line with the grain multi-chain scenario is designed to realize the collaborative consensus and management of the whole process information.

The rest of this paper is organized as follows. Section 2 presents the preliminary work and clarifies the contributions of this paper. Section 3 designs a multi-chain blockchain model for grain food based on zero-knowledge proof, including information deconstruction of grain food supply chain, multi-chain network model design, privacy hierarchical encryption and storage design, cross-chain communication model design, and whole consensus algorithm design. Section 4 analyzes and verifies the overall performance of the overall model and the performance of the consensus algorithm and verifies the theoretical model. Section 5 presents the conclusions of this paper and reflects on the insufficiency of the study.

## 2. Literature Review

Blockchain is a new generation of information technology originated from digital cryptocurrency. Broadly speaking, blockchain technology is a decentralized infrastructure and distributed computing paradigm that verifies and stores data through an encrypted chain data structure that generates and updates data through a distributed node consensus algorithm. In addition, blockchain technology programs and operates data through a smart contract, and ensures data transmission and access security through an encryption algorithm. It has been widely used in finance, medical treatment, notarization, commerce, government affairs, supply chain, food safety, and other fields in recent years [24]. Blockchain, the characteristics of decentralization, time series data, collective maintenance, programmability, and safety and reliability, are well suited to agricultural products and food supply chain systems [25]. The mainstream consensus algorithms currently used in agricultural products and food include PBFT [26], Raft [27], Kafka [28], and other consensus algorithms and related optimizations, but few consensus algorithms combined with zero-knowledge proof have been studied.

The research on the construction of a grain multi-chain blockchain based on a zero-knowledge proof has higher requirements of the information data security of the whole grain supply chain, the real-time interaction among chains, and the ability of the whole chain’s information collaboration. In this section, we collect some frontier research on the application of blockchain technology in agricultural products and food, as shown in Table 1.

Agricultural products and food research based on single-chain models mainly focus on three dimensions of applied theoretical research, data application research, and data supervision research. Traditional agricultural products and food supply chains have problems in terms of food quality safety, food authenticity, and circulation efficiency. The application of blockchain technology can effectively enhance the application and management of supply chain data [29,30]. Although in the supply chain scenario, there is great potential in the application of blockchain in data volume and transaction speed, it also faces challenges such as data ownership and data operability in the process of digital transformation [31,32]. Blockchain technology can effectively improve and apply informationization to agricultural products and food supply chains. However, the complex characteristics of the grain food supply chain pose many challenges to the application of blockchain technology. 

In terms of data application research based on the traceability of blockchain technology, Refs. [33,34,36] built a traceability system framework applicable to agricultural products and food supply chain based on the decentralized and traceable characteristics of blockchain, which defuses the risks of the traditional centralized mode, providing transparent data for enterprises and bringing reliable traceability information to consumers. One study [35] achieved the trusted traceability of the soybean supply chain with smart contract technology. The design of the smart contract eliminated the dependence on trusted third parties, which not only provided decentralized and trusted traceability but also completed the interactive management of all participants through the contract, providing an idea for the supervision of agricultural products and the food supply chain.

In terms of research on data control of tamper-proof characteristics based on blockchain, Ref. [37] proposed a food market regulation model method based on blockchain and deep learning-SAE, which controls the risk by analyzing consumers’ emotional tendencies to understand their needs. It is crucial to establish consumers’ confidence in food safety. The research in [15] built a blockchain-based information security supervision model for agricultural products and food supply chains. By optimizing the complex information flow management of the supply chain through smart contracts, the accuracy, efficiency, and credibility of the supervision process can be achieved. Ref. [38] proposed a credible management scheme for strategic grain reserves based on blockchain technology. Blockchain can provide improved management and transparent governance capabilities, which has a bright future in grain supervision. Through the analysis of the above references, we can see that researchers have conducted several studies on agricultural products and food supply chains from a multi-dimensional perspective, but these studies are all based on a single-chain architecture. Facing the huge amount of data and complex business relationships in the grain food supply chain poses a severe test on the information storage, consensus verification, and data processing capacity of the blockchain.

With the development of multi-chain blockchain models in recent years, some researchers have explored the application of multi-chain technology in agricultural products and food supply chains. Ref. [39] proposes a food safety supervision system HMDBC based on the hierarchical multi-domain blockchain network architecture, which divides the upper main chain network and the lower multi-sub-chain network. The main chain and sub-chains are in a mapping relationship, which has more advantages in traceability and storage capacity compared with the traditional single chain. Ref. [20] proposed a trusted traceability model for the whole grain and oil food supply chain based on trusted blockchain and trusted identification and designed a master–slave multi-chain storage model and trusted identification mechanism, which effectively solved the problems of cluttered information storage, low traceability security and poor data sharing in the supply chain, providing ideas for the application of information among multiple chains. From the perspective of information collaboration, Ref. [21] constructed a rice supply chain information management and control model based on multi-chain collaboration and realized the multi-chain collaboration management and control of rice supply chain information through the design of the trusted chain mechanism, multi-level sub-chain encryption mechanism, trusted supervision mechanism and hierarchical consensus mechanism. In order to solve the complex information supervision problem in the rice supply chain, Ref. [22] constructed a data cross-chain supervision model based on parallel blockchain and smart contracts. By designing information collection/supervision cross-chain mechanism, data concurrency mechanism and hierarchical consensus mechanism, high availability and security of rice supply chain information interaction and control are achieved. Ref. [40] proposes an agricultural supply chain system architecture based on a double-chain structure and designs a consensus algorithm for the agricultural enterprise resource blockchain based on POS, which is more concise and considers more of the weights. The proposed system can better take into account the transparency, security and privacy of the transaction. Through the analysis of the above references, we can see that those studies mainly focus on building a multi-chain architecture suitable for agricultural products and food supply chains but do not focus on the privacy protection of data information, access rights management and global information collaborative consensus in multi-chain scenarios. The above issues will be the focus of this paper in the grain food application scenario.

A consensus algorithm is the core element in blockchain technology and is the consistency protocol for coordinating distributed networks under the blockchain network model without central control. Ref. [11] proposed a CPBFT consensus algorithm based on credit evaluation on the basis of designing a rice supply chain information supervision model, which greatly reduced the probability of failure node hosting and participating in consensus under Byzantine fault-tolerant algorithm, and further improves the consensus efficiency in rice scenarios. Ref. [41] analyzed the integration framework of the combination of blockchain and supply chain and proposed a parallel practical Byzantine fault-tolerant consensus algorithm based on PBFT. This scheme is significantly effective for solving the low consistency problem caused by the rapid expansion of nodes in the supply chain. Ref. [27] proposed a halal food supply chain management scheme based on Hyperledger multi-channel technology and adopted a Raft consensus to realize the data information consistency authentication of three channels. Ref. [28] proposed a permissioned, chain-based platform Rahasak to improve the scalability of the blockchain and used Kafka consensus to improve the ability to process transactions concurrently under high transaction throughput. The research on the consensus algorithm of agricultural products and food supply chains in the above references ensures the consistency and security of information under a single-chain structure, but there is still a lack of a complete consensus mechanism to achieve information collaboration, consistency verification, and information security under the entire supply chain and multiple chains.

A zero-knowledge proof includes two modes: interactive and non-interactive. Interactive zero-knowledge proof requires both parties to communicate through several rounds of interaction to achieve the proof effect. However, in the scenario of many participating nodes in the blockchain, the frequent interaction of multiple participants will cause network delays, DoS (denial of service), and other problems. Therefore, non-interactive zero-knowledge proofs are generally used in the blockchain scenario [46]. At present, the application of a zero-knowledge proof combined with blockchain technology mainly focuses on identity authentication and privacy protection. Ref. [42] proposes an identity authentication model that combines blockchain and zero-knowledge proof. The privacy attribute token secretly performs privacy attribute ownership and identity ownership authentication, realizing identity unlinkability and behavioral privacy. Refs. [43,44] proposes a privacy protection scheme based on zero-knowledge proof. Since no information will be revealed to the verifier during the verification, the data integrity and privacy protection of the proof process can be realized. By referring to traditional consensus mechanisms as well as the randomicity and irreversibility of quantum measurement and quantum zero-knowledge proof, Ref. [45] proposed a quantum blockchain zero-knowledge consensus with lower demand in computing resources, shorter delay and higher throughput, which also provides a feasible idea for this paper. Through the above analysis, we know that a zero-knowledge proof has great potential for privacy protection. Its application to the consensus level can effectively realize the secure collaborative consensus of privacy data in the chain without revealing any relevant information about privacy data. Therefore, this paper will construct a zero-knowledge consensus by adopting the typical algorithm Groth 16 protocol in zk-SNARK, the protocol of which has been relatively mature in the application of Zcash and Feilcoin projects.

Based on the comprehensive analysis of the above studies, the specific contributions of this paper are as follows:

- The paper analyzes the information characteristics of the whole life cycle of the grain supply chain comprehensively, classifies the key information of each link into consideration of actual business scenarios, and designs a multi-chain-based grain supply chain network model to achieve data isolation in each link and reduce redundant data storage of chains.
- The hierarchical encryption and storage mode of privacy data is designed for different levels of privacy information, which realizes the differentiated management and application of various types of data. In addition, a cross-chain communication mode based on the relay chain is designed to realize the business interaction of each chain, and the data integrity is effectively guaranteed through digital signature and smart contract technology.
- A consensus mechanism based on PBFT and the zero-knowledge proof is designed, including three steps: CPBFT consensus on the business chain, ZKP consensus on the relay chain, and KZKP consensus on the consensus chain, which realizes the multi-chain information collaborative consensus of the grain food supply chain, and ensures the information coordination in the whole chain from the information level.

## 3. Construction of Grain Food Multi-Chain Blockchain Model Based on Zero-Knowledge Proof

### 3.1. Information Deconstruction

The grain food supply chain has the characteristics of complex participation links, a long life cycle, and multiple participants. The process involves planting, purchasing, warehousing, processing, transportation, and sales enterprises. Different permission information within each enterprise can be viewed by personnel with different positions and authority. There is also specific business information interaction between upstream and downstream enterprises. Consumers have requirements for grain traceability information, and regulatory authorities have requirements for specific regulatory information. Therefore, facing the complicated business information of the grain food supply chain, this section analyzes the characteristics of various types of information in different links by category, summarizes the specific contents of various types of information in each link, and classifies the key information according to the authority, providing support for enterprises to carry out differentiated management and sharing of key information.

#### 3.1.1. Information Analysis of Key Links in Grain Food Supply Chain

**Figure 1:**
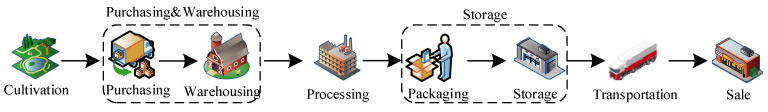
Grain food supply chain link process.

As shown in Figure 1, the whole grain food supply chain links include six major links: planting, purchasing, processing, storage, transportation, and sales. The storage link includes two steps of purchasing and warehouse storage, and the storage link includes two steps of packaging and storage. By comprehensively analyzing the business process of each link and the characteristics of participating enterprises, the key information of the six links and eight steps are divided into four subcategories, namely basic information, environmental information, hazard information and transaction information, as shown in Appendix A Table A1. Among them, the basic information mainly includes the operation information, time information, product information and quality information of the corresponding link. The environmental information includes the environmental conditions of raw grains, semi-finished products and finished products. The hazard information includes mycotoxins, pesticide residues, heavy metals, pests and diseases and quality deterioration-related information involved in raw grain, semi-finished products and finished products. The transaction information mainly involves product-related price and cost information. All kinds of information involve different degrees of privacy information, which is not suitable for all disclosure. The key information is classified by authority, which establishes the foundation for classified encryption of privacy information and the construction of multi-chain network architecture.

#### 3.1.2. Privacy Information Authority Classification

By analyzing the key information of each link of the grain supply chain, the key information is divided into level I privacy information, level II privacy information, level III privacy information, public information, and regulatory information, according to the authority, as shown in Appendix A, Table A2. Level I privacy information is the data that can be accessed with the highest authority within the enterprise. Level II privacy information is the data accessible to other employees within the enterprise. Level III privacy information is the data that can be accessed among upstream and downstream production enterprises. Public information refers to consumer-oriented traceability information. Regulatory information refers to the key information that the regulatory authorities supervise the upstream and downstream enterprises in real-time to ensure the safety of production links. All levels of privacy authority are downward compatible. Since the regulatory authority exists independently from the enterprise, and the regulatory information intersects with privacy information and public information, the authority of the regulatory authority is independent of the authority of privacy information.

### 3.2. Information Deconstruction

#### 3.2.1. Information Analysis of Key Links in Grain Food Supply Chain

**Figure 2:**
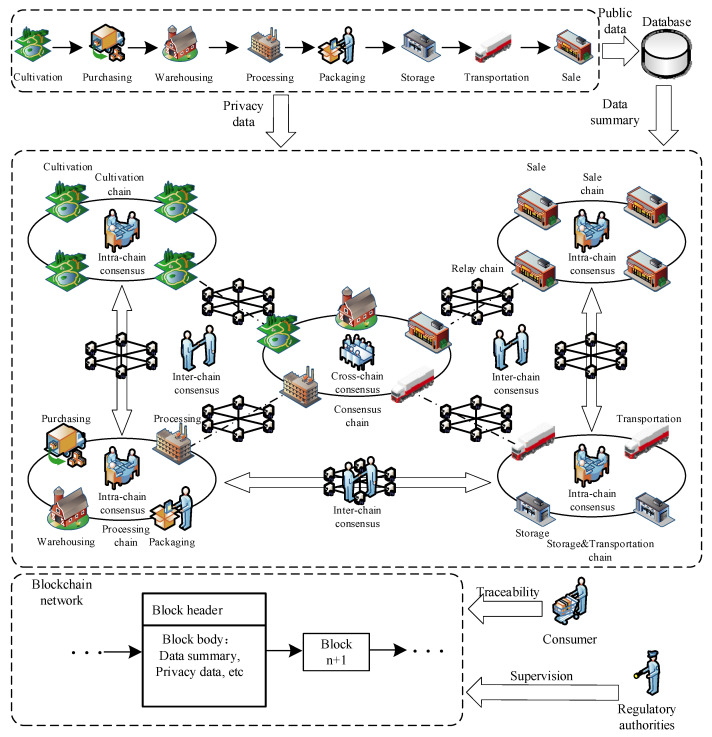
Multi-chain network model of grain food supply chain.

The grain food supply chain has the characteristics of complex participation links, a long life cycle, and many participating companies. Though traditional single-chain structure solves the problem of data decentralization to some extent, it is of no use to deal with the data redundancy and differentiated data sharing and storage caused by the grain food supply chain, nor ease the storage burden caused by the business growth. This section builds a multi-chain network model of the entire grain supply chain, combined with the multi-chain and relay cross-chain mechanisms according to the process information flow characteristics of the grain supply chain and the business logic of the enterprises in each link. The multi-chain network structure model is shown in Figure 2.

Based on the actual business scenario, the six major links of the grain supply chain are divided into four business chains, each key link with business associations corresponding to a sub-chain, namely the planting chain, the processing chain, the storage and transportation chain, and the sales chain. In addition, there is a consensus chain responsible for completing the cross-chain consensus of the entire supply chain. Data are exchanged through the relay chain. Each chain corresponds to the corresponding business link nodes, and the consensus chain is composed of planting, warehousing, processing, storage, and transportation and sales nodes. Each business chain completes the intra-chain consensus, and each business chain completes the data interaction through the relay chain, and the inter-chain consensus step is completed by the relay chain. When the encrypted data of the business chain is uploaded to the consensus chain, the consensus chain completes the cross-chain consensus step of the supply chain so as to complete a round of information cross-chain consensus of the whole link. The privacy information entity is stored in the block structure of the business chain. The public information entity is stored in the off-chain database, while the data abstract is stored in the on-chain database. Consumers can check the corresponding public tracing information by referring to the business chain and verifying if it has been tampered with, and the regulatory authority can conduct real-time supervision of enterprises. On the basis of solving data redundancy of the single-chain blockchain, this framework achieves the data isolation of each business, laying a foundation for key information differentiated management with differentiated degrees of privacy in each link.

#### 3.2.2. Hierarchical Encryption and Storage Mode of Privacy Data

**Figure 3:**
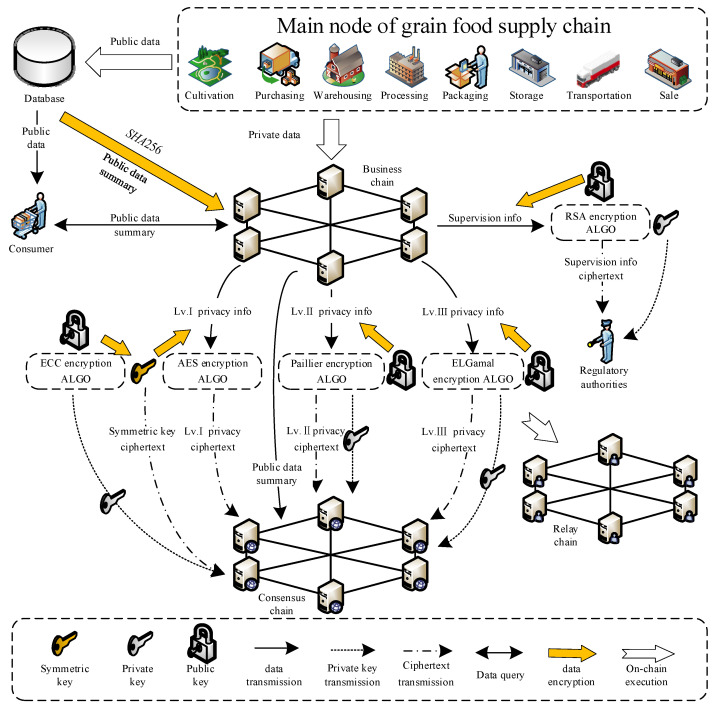
Hierarchical encryption and storage mode of private data.

In the previous section, a multi-chain network model of the grain food supply chain is constructed. Each business chain stores its own business privacy data through the blockchain’s data structure. However, the data interaction, communication and consensus among chains have brought new risks and challenges to privacy data, so it is critical to encrypt privacy data. To ensure the security of different degrees of privacy data in the process of transmission of the food supply chain, the hierarchical encryption and storage mode of privacy data is designed based on asymmetric encryption and symmetric encryption cryptography, as is shown in Figure 3. During the grain digital transformation, research studies in this paper are mainly targeted at standardized grain and oil enterprises in the food industry. System parameters will be preset by the staff only during the system initialization, the process of which is subject to local laws and regulations and business norms to ensure the authenticity of the human recording. During the system cycle operation, the data information involved in the grain supply chain is totally collected by RFID, GPS, camera, sensor, QR code scanner, and other equipment. The amount of public information data is relatively complex, which is stored in the off-chain database, and its abstract is uploaded to the business chain. The plain text of privacy information is stored in the business chain through the hash tree structure of the blockchain to ensure data security. The data interaction and encryption among the business chain and other chains are completed through the relay chain. The hierarchical encryption method is adopted according to different privacy levels. The relay chain, as the intermediary of information transmission, does not store any information.

According to the authority classification of privacy information, level I privacy information is the data that can be accessed by the highest authority within the enterprise with a moderate amount of data. Level II privacy information is the data that can be accessed by other employees within the enterprise with a relatively large amount of data. Level III privacy information is the data that can be accessed by each other among upstream and downstream production enterprises with a moderate amount of data. Public information is consumer-oriented traceability information, which is relatively complex. Regulatory information is the key information for regulatory authorities to supervise upstream and downstream enterprises in real-time to ensure the safety of production links, and the authority is independent of that of privacy information. In order to ensure the security and privacy of the data privacy encryption process, some of the keys and random parameters in this section are generated by the Reverse Method based on a pseudo-random number generator to ensure that the parameters have sufficient randomness, aperiodicity, and unpredictability. In the selection process of random large prime numbers, first, select a large random number and then determine whether it is a prime number. The encryption process for these five types of information is as follows.

1. 1. Level I privacy information;

Level I privacy information adopts AES and ECC hybrid encryption [47]. Since symmetric encryption and decryption use the same key, the fast encryption and decryption feature is applicable to a large amount of data. However, in the process of channel transmission, once the key is leaked, the ciphertext has the risk of cracking and public tampering. Therefore, the asymmetric encryption algorithm ECC is used to encrypt the symmetric encryption algorithm AES key. The specific process is as follows.

First is the hybrid encryption process, which uses the AES encryption algorithm ECB mode to encrypt the plaintext of the data. The encryption process is as shown in Equation (1). *m*_I_ is the plaintext of level I privacy information, *k_A_* is the symmetric encryption key acquired through KeyExpansion, and *c*_I_ is the ciphertext of level I privacy information.

(1) $$c_{I}=Enc_{AES}(m_{I},k_{A})$$

Suppose *F_p_* is a finite field, *p*_I_ is the feature of *F_p_* and is a safe large prime. *E* is an elliptic curve determined according to the equation $y^{2}=x^{3}+a_{1}x^{2}+b_{1}$ which satisfies $4a_{1}^{2}+27b_{1}^{2}\neq 0$, *G* is the base point of *E*, *n*_I_ is the order of *F_p_*. The receiver chooses a large prime number *k*_I_ as the private key randomly, (*k*_I_ < *n*_I_*)*. By calculating the public key *K*_I_ through the point multiplication operation of large numbers of the elliptic curve *E* in Equation (2), the receiver sends (*G*, *K*_I_) to the symmetric key sender. The sender encodes the symmetric encryption algorithm key *k_A_* to a point on the elliptic curve through BCH. Select an integer *r*_I_ less than *n*_I_ randomly, encrypt the transmitted point M through Equations (3) and (4), then the sender sends (*C*_1_, *C*_2_, *c*_I_) to the receiver.

(2) $$K_{I}=k_{I}*G$$

(3) $$C_{1}=M+r_{I}K_{I}$$

(4) $$C_{2}=r_{I}G$$

Then, in the decryption process, the receiver receives (*C*_1_, *C*_2_, *c*_I_) and uses the private key *k*_I_ to decrypt the point M by Equation (5). Decode point M to obtain the AES symmetric encryption key. Then, use Equation (6) to decrypt and restore the ciphertext of the level I privacy data to obtain the plaintext of the data.

(5) $$C_{1}-k_{I}C_{2}=M+r_{I}K_{I}-k_{I}(r_{I}G)=M+r_{I}(k_{I}G)-k_{I}(r_{I}G)=M$$

(6) $$m_{I}=Dec_{AES}(c_{I},k_{A})$$

During the transmission, the level I privacy information is transmitted in the form of ciphertext, and the symmetric encryption key is encrypted by the ECC encryption algorithm. Even if *C*_1_, *C*_2_ or point *G*, *K*_I_ is intercepted during the transmission, the receiver’s private key *k*_I_ cannot be deduced due to the elliptic curve discrete logarithm problem, and the symmetric encryption key *k_A_* cannot be deduced, thus ensuring the security of the data transmission.

2. Level II privacy information

Level II privacy information adopts Paillier homomorphic encryption. Through the homomorphic characteristics of the algorithm, the ciphertext can be directly operated, and the plaintext result of the corresponding operation can be obtained after decryption. The specific process is as follows.

During the key generation, select two different large prime numbers *p*_II_ and *q*_II_ randomly, and make sure the gcd of *p*_II_*q*_II_ and (*p*_II_ − 1)(*q*_II_ − 1) is 1. The product of *p*_II_ and *q*_II_ is *n*_II_, and the lcm of (*p*_II_ − 1) and (*q*_II_ − 1) is *λ*_II_. Choose a positive integer *g*_II_ randomly, $g_{\mathrm{II}}\in Z_{n_{\mathrm{II}}^{2}}^{*}$, and *g*_II_ is less than $n_{\mathrm{II}}^{2}$ and coprime to $n_{\mathrm{II}}^{2}$. Define *L*(*x*) to represent function (*x* − 1)/*n*_II_. *μ*_II_ can be calculated by Equation (7), then (*n*_II_, *g*_II_) is the public key and (*λ*_II_, *μ*_II_) is the private key.

(7) $$\mu_{\mathrm{II}}={\left[L(g_{\mathrm{II}}^{\lambda_{\mathrm{II}}}\mathrm{mod}n_{\mathrm{II}}^{2})\right]}^{-1}\mathrm{mod}n_{\mathrm{II}}$$

During the encryption, for the plaintext of level II privacy data, *m*_II_ is cut into k segments, and the cut data is $m_{\mathrm{II}}{}_{1},\ldots ,m_{\mathrm{II}}{}_{k}$, $0\leq m_{\mathrm{II}i}<n_{\mathrm{II}}(i=1,2\ldots k)$. Choose an integer *r*_I_ randomly, $r_{i}\in Z_{n_{\mathrm{II}}^{2}}^{*}$ and $r_{i}<n_{\mathrm{II}}$. Use the public key (*n*_II_, *g*_II_) to encrypt the plaintext segments respectively. The encryption process is as shown in Equation (8), and the encrypted data $c_{\mathrm{II}1},\ldots ,c_{\mathrm{II}k}$ is obtained.

(8) $$c_{\mathrm{II}i}=E(m_{\mathrm{II}}{}_{i},r_{i})=g_{\mathrm{II}}^{m_{\mathrm{II}i}}r_{i}^{n_{\mathrm{II}}}\mathrm{mod}n_{\mathrm{II}}^{2}$$

During the decryption, the private key (*λ*_II_, *μ*_II_) is used to decrypt the ciphertext *c*_II_ of level II privacy data. The decryption process is as in Equation (9), and the plaintext data segment $m_{\mathrm{II}}{}_{1},\ldots ,m_{\mathrm{II}}{}_{k}$ is obtained. Then, the plaintext of level II privacy information can be obtained by Equation (10).

(9) $$m_{\mathrm{II}i}=D(c_{\mathrm{II}i})=L(c_{\mathrm{II}i}^{\lambda_{\mathrm{II}}}\mathrm{mod}n_{\mathrm{II}}^{2})\cdot \mu_{\mathrm{II}}\mathrm{mod}n_{\mathrm{II}}$$

(10) $$m_{\mathrm{II}}=\sum_{i=1}^{k}m_{\mathrm{II}i}$$

(11) $$D[\prod_{i=1}^{k}E(m_{\mathrm{II}i},r_{i})\mathrm{mod}n_{\mathrm{II}}^{2}]=D[g^{\sum_{i=1}^{k}m_{\mathrm{II}i}}\cdot {\left(\prod_{i=1}^{k}r_{i}\right)}^{n_{\mathrm{II}}}\mathrm{mod}n_{\mathrm{II}}^{2}]=\sum_{i=1}^{k}m_{\mathrm{II}i}\mathrm{mod}n_{\mathrm{II}}$$

Due to the homomorphic property of addition, the result obtained by multiplying the segmented ciphertext after cutting and then decrypting is the same as that by directly adding the segmented plaintext, as shown in Equation (11). Therefore, the ciphertext of the private data can be obtained without knowing the plaintext segment of the private data, and the encryption efficiency can be improved by performing parallel computing on the data segment.

3. Level III privacy information

Level III privacy information adopts ElGamal homomorphic encryption. ElGamal is a public key cryptosystem based on discrete logarithm problems over finite fields, and its security is based on the difficulty of solving discrete logarithms [48]. Its operation speed is affected by the key length, and it is suitable for level III privacy information scenarios that do not require high data real-time performance. The specific process is as follows.

During the key generation, suppose $G_{Z_{p}}$ is a multiplicative group of finite field *Z_p_*. Randomly generate a large prime number *p*_III_ and select the generator *g*_III_, $g_{\mathrm{III}}\in Z_{p}^{*}$. Randomly select $k_{\mathrm{III}}\in [1,p_{\mathrm{III}}-1]$ as the private key. Calculate the public key by Equation (12), disclose the public key (*y*_III_, *g*_III_, *p*_III_), and save the private key *k*_III_.

(12) $$y_{\mathrm{III}}=g_{\mathrm{III}}^{k_{\mathrm{III}}}\mathrm{mod}p_{\mathrm{III}}$$

During the encryption, the data sender selects a random number *r*_III_, $r_{\mathrm{III}}\in [1,p_{\mathrm{III}}-1]$. Use the system parameters and public key *y*_III_ to encrypt the level III privacy data *m*_III_ and calculate the level III privacy data ciphertext by Equations (13)–(15).

(13) $$C_{3}=g_{\mathrm{III}}^{r_{\mathrm{III}}}\mathrm{mod}p_{\mathrm{III}}$$

(14) $$C_{4}=m_{\mathrm{III}}y_{{}_{\mathrm{III}}}^{r_{\mathrm{III}}}\mathrm{mod}p_{\mathrm{III}}$$

(15) $$c_{\mathrm{III}}=E(m_{\mathrm{III}})=(C_{3},C_{4})$$

During the decryption, after receiving the ciphertext *c*_III_, the receiver uses the private key *k*_III_ to decrypt it and calculates the level III privacy data plaintext by Equation (16).

(16) $$m_{\mathrm{III}}=D(E(m_{\mathrm{III}}))=\frac{C_{4}}{C_{3}^{k_{\mathrm{III}}}}\mathrm{mod}p_{\mathrm{III}}$$

In the ElGamal cryptosystem, the encryption operation is random, and the ciphertext depends on both the plaintext and the random private key, so there may be *p*_III_ − 1 possible ciphertexts for the same key.

4. The supervision information

The supervision information is encrypted by RSA. Since the supervision information overlaps with various types of information and its authority are relatively independent, the RSA algorithm with high security is used for encryption. The specific process is as follows.

During the key generation, two large different prime numbers *p_s_* and *q_s_* are randomly generated. (*n_s_*, φ(*n_s_*)) is calculated according to Equations (17) and (18), in which *n_s_* is the modulus and φ(*n_s_*) is the Euler’s totient function. A positive integer *e_s_* is selected randomly that satisfies $1<e_{s}<\phi (n_{s})$, gcd *e_s_*, and φ(*n_s_*) is 1. The modular inverse element *d_s_* is calculated according to Equation (19).

(17) $$n_{s}=p_{s}q_{s}$$

(18) $$\phi (n_{s})=(p_{s}-1)(q_{s}-1)$$

(19) $$d_{s}=e_{s}^{-1}\mathrm{mod}\phi (n_{s})$$

The public key (*n_s_*, *e_s_*) and private key (*n_s_*, *d_s_*) are obtained. The public key, which is distributed to data senders, is responsible for encrypting the supervision information and the private key is distributed to the regulation department to decrypt and destroy (*p_s_*, *q_s_*,φ(*n_s_*)). During the encryption, the plaintext of the supervision data is encrypted using the public key according to Equation (20). After receiving the ciphertext, the regulation department uses the private key (*n_s_*, *d_s_*) to decrypt the plaintext of the supervision data according to Equation (21).

(20) $$c_{s}=E(m_{s})=m^{e_{s}}\mathrm{mod}n_{s}$$

(21) $$m_{s}=D(c_{s})=c_{s}^{d_{s}}\mathrm{mod}n_{s}$$

After generating the system key, the generated parameters are destroyed so that the key and related parameters cannot be decomposed. The difficulty of decomposing large prime numbers ensures the transmission security of supervision data.

5. The public information

The public information adopts SHA256 to extract the abstract of the information subject. The public information subject is stored in the off-chain database, and the public information is recorded in the form of data abstracts on and between chains. The public information is compressed in plaintext by SHA256 of the SHA-2 family, which reduces the amount of data stored and ensures the security of the data during transmission.

(22) $$H_{m}=Hash(m_{pub})$$

*m_pub_* is the plaintext of the public information. The public information data digest *H_m_* is calculated according to Equation (22), in which the *Hash*(*x*) is the hash function SHA256, and a hexadecimal data digest string with a length of 64 is obtained after compressing. All links in the grain food supply chain, including consumers and regulatory authorities, have access to public information and can verify whether public information has been tampered with through data digests.

#### 3.2.3. Relay Cross-Chain Communication Mode

A multi-chain-based grain food scene information model has been constructed in the previous study, which effectively solves the problems of single-chain data storage redundancy, differential information sharing and authority management. However, this also introduces new systemic challenges. Enterprise chains with business connections are no longer connected to each other, forming a kind of ‘information island’. An effective interaction mechanism is needed between chains to realize cross-chain data transmission, solve the problems of data cross-chain communication and trusted communication, and provide a certain amount of computing power for the main chain to improve inter-chain interoperability.

As a combination of the side chain and notary mechanism, the relay chain has the decentralization feature of accessing and verifying the key information of the two chains for interoperability and transferring the cross-chain information of the two chains. In the relay mechanism, the business chain is connected to the relay chain by complying with the protocol specifications. When the business chain needs to initiate a cross-chain operation, it will publish the data information that needs to perform the cross-chain operation after passing the verification and review of the relay chain. The relay chain transfers information to the target business chain or consensus chain to realize cross-chain operation.

**Figure 4:**
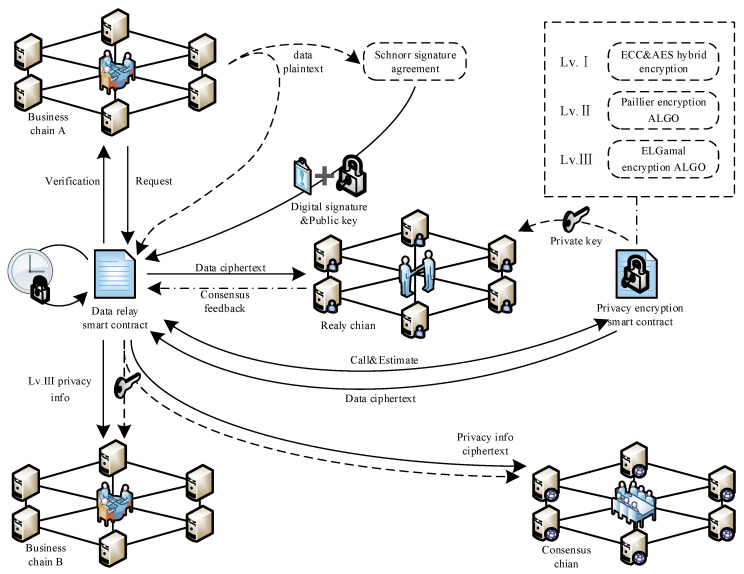
Relay cross-chain mode.

The relay cross-chain mode is shown in Figure 4. The specific relay process is as follows.

When the two chains need to perform cross-chain communication and interaction, the DRSC (Data Relay Smart Contract, as shown in Appendix A, Algorithm A2) will monitor the cross-chain request in the network. Business chain A sends a request to the DRSC, and the contract verifies the request and forwards the request to the corresponding receiving chain. The verification includes the sender, the receiver, and the cross-chain sequence. After passing the verification, a communication channel from business chain A to the relay chain, and then to business chain B or the consensus chain is created. This process only occurs when cross-chain information exchange is required. After the two chains successfully communicate through the relay chain, any request for data exchange between chains will return an exception report.

After the request is verified, the Schnorr Signature Protocol is used to digitally sign the data plaintext to be transmitted. The same elliptic curve *E* determined by the Weierstras equation is used for hybrid encryption of level I privacy information, and *G* is the base point on the curve. A prime number *k_sIg_* is selected as the private key *sk_sIg_* randomly, then the elliptic curve point multiplication k_sIg_*G as the public key *pk_sIg_*, which is assigned to the DRSC for verification. A random number, *r_sIg_*, is randomly selected. The business chain calculates *R_sig_*, signature *c_sig_*, and *z_sig_*, respectively, through Equations (23)–(25). Then, the business chain sends the signature (*c_sig_*, *z_sig_*) and the data plaintext *m_sig_* to the DRSC.

(23) $$R_{sig}=r_{sig}*G$$

(24) $$c_{sig}=Hash(m_{sig},R_{sig})$$

(25) $$z_{sig}=r_{sig}+c_{sig}*sk$$

(26) $$R_{{}_{sig}}^{\prime }=z_{sig}*G-c_{sig}*pk$$

The DRSC performs the verification process after receiving the signature. Calculate $R_{{}_{sig}}^{\prime }$ by Equation (26) and judge whether *c_sig_* is equal to $Hash(m_{sig},R_{{}_{sig}}^{\prime })$. If the equation holds, it is confirmed that the message has not been tampered with during the transmission for hierarchical permission encryption; otherwise, the cross-chain information transmission process is terminated.

After judging the integrity of the data, judge the authority level of the transmitted data in plaintext by calling the PESC (Privacy Encryption Smart Contract, as shown in Appendix A, Algorithm A1) is needed. If it is the cross-chain interaction of level III privacy information between the upstream and downstream enterprise business chains, the ElGamal encryption algorithm should be used to encrypt the data. The private key is sent to the relay chain, and the ciphertext is sent to the DRSC and the relay to the relay chain. The relay chain achieves the consensus of business interaction data between upstream and downstream enterprise chains through ZKP (Zero-knowledge Proof based consensus algorithm) step. In the process of transferring shared data to the consensus chain, the food supply chain public data digest and the privacy data in each business chain blockchain is transmitted in the form of a hash and must be completed by the data relay smart contract after the cross-chain consensus. After the relay chain reaches a consensus between the business chains, the DRSC sends the shared data and private key to the receiver of the service link. If the consensus process fails, the relay process is interrupted. If it is the data interaction between the business chain and the consensus chain, the privacy level of the plaintext data is judged by calling the PESC, and different levels of encryption methods are adopted according to different permissions, the data feedback to the DRSC. The private key is sent to the relay chain, and the DRSC sends the ciphertext data to the relay chain to complete the inter-chain consensus process. The encrypted ciphertext and private key are transmitted to the consensus chain after the relay chain comes to a consensus. The consensus chain completes the multi-chain and cross-chain consensus process by KZKP (Kafka-based Zero-knowledge Proof consensus algorithm) step. Time lock T is designed in DRSC. If more than 51% of the nodes in the business chain or the consensus chain have received the data packets sent from the relay chain, the relay chain is deemed to have completed the cross-chain data transmission, destroy the storage of data ciphertext within time T. This mechanism ensures the security of data cross-chain interaction of food supply chain through combining relay chain, smart contracts, and digital signature, as well as through the signature verification, encryption transmission and zero-knowledge consensus of all data in the grain food supply chain.

### 3.3. Zero-Knowledge Proof-Based Consensus Mechanism

During the data transmission in the food supply chain, there are problems, such as lower efficiency in the system consensus caused by potential vicious nodes within business chains, the cross-chain trusted interaction verification, as well as how to achieve the verification that is available but not visible in the whole food supply chain. To solve these problems, this section proposes a consensus mechanism based on zero-knowledge proof, which mainly includes three steps: Step 1 is the CPBFT consensus on the business chain, step 2 is the consensus based on zero-knowledge proof on the relay chain, and step 3 is the zero-knowledge proof consensus based on Kafka on the consensus chain, all of which together form a multi-chain collaborative consensus.

#### 3.3.1. CPBFT Consensus Mechanism

The PBFT algorithm provides a Byzantine fault tolerance rate of 1/3 of the total number of nodes in the system. However, in the grain food supply chain, there are many participating nodes, and the possibility of Byzantine nodes also increases. When the master node behaves maliciously, all nodes need to be re-elected for view replacement, resulting in the low consensus efficiency of the system. Malicious nodes affect the efficiency of the algorithm from multiple dimensions. In order to reduce the impact of malicious nodes on the algorithm, this section designs a credit practical Byzantine Fault Tolerant CPBFT (Credit Practical Byzantine Fault Tolerant consensus algorithm) suitable for the whole grain food supply chain. The node credit evaluation mechanism is first introduced to evaluate the credit of the nodes in the system, and the nodes in the system are divided into master nodes, candidate nodes, ordinary nodes and faulty nodes according to the trust threshold. At the same time, the candidate queue of the master node is set to reduce the probability of the failed node participating in the election as the master node. Then, in order to reduce the communication complexity among nodes, the consensus protocol of the PBFT algorithm is simplified, which further improves the consensus efficiency.

6. Node credit evaluation mechanism.
  (a) Initial credit value setting

**Figure 5:**
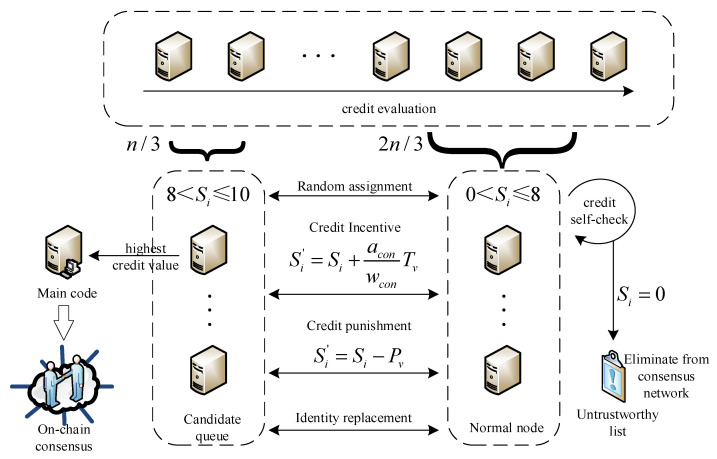
Node credit evaluation mechanism of CPBFT.

With the increase of participating nodes in the grain food supply chain, the probability of faulty nodes also rises. Taking into account the scale and social reputation of participating enterprises, the credibility of participating enterprises is ranked quantitatively. And take the first n/3 nodes as candidate nodes, *S_i_* represents the credit value of node *i*, then assign credit values to nodes randomly. The credit value of the first n/3 nodes is assigned in interval $8\;<\;S_{i}\;\leq \;10$, and the credit value of the last 2n/3 nodes is assigned in interval $0\;<\;S_{i}\;\leq \;8$, the credit value is accurate to two decimal places. As shown in Figure 5, nodes with credit values between 8 and 10 are candidate nodes. During the view change, the candidate node with the highest credit value is selected as the master node. When multiple nodes have the same highest credit values after several iterations, one node is randomly selected as the master node to hold the consensus of the next round. When the credit value meets $0\;<\;S_{i}\;\leq \;8$, nodes participate in the system consensus process as ordinary nodes. When the credit value meets *S_i_* = 0, it is indicated that the node is a malicious node with multiple failures and is included in the list of untrustworthy nodes and excluded from the consensus network.

  (b) Credit value reward and punishment mechanism

Several rounds of consensus process are performed, and the faulty nodes that exist in the consensus process or the nodes that send messages inconsistent with most nodes are eliminated. In order to encourage each node to participate in the consensus honestly and resist faulty nodes, a reward and punishment mechanism for node credit value is designed. The checkpoint protocol updates the credit value of each node through the log to avoid malicious propagation and attempts to disrupt the consensus process.

The credit value reward mechanism evaluates the number of successful communications when nodes participate in the process. The credit value reward mechanism evaluates the number of successful communication times when nodes participate in the process. $S_{i}^{\prime }$ is the updated credit value. *T_v_* is the reward value adjustment parameter, *w_con_* is the expected number of consensus participants, *a_con_* is the actual number of consensus participations, and the credit value reward mechanism is shown in Equation (27).

(27) $$S_{i}^{\prime }=\left\{ \begin{array}{l} S_{i}+\frac{a_{con}}{w_{con}}T_{v1},\;0\;<\;S_{i}\;\leq \;8\\ S_{i}+\frac{a_{con}}{w_{con}}T_{v2},\;8\;<\;S_{i}\;\leq \;10 \end{array}\right.$$

When the candidate nodes successfully complete a round of consensus, the ratio of *a_con_* to *w_con_* is 1. By setting $T_{v1}\;>\;T_{v2}$, all nodes are encouraged to complete the consensus process honestly. More credit rewards make ordinary nodes more likely to join the queue of candidate nodes and become the master node to host the view.

The credit value punishment mechanism is mainly used to regulate the credit value of the failed node. During the consensus, the nodes did not communicate successfully. Under this condition, *a_con_* = 0. The punishment mechanism is shown in Equation (28).

(28) $$S_{i}^{\prime }=\left\{ \begin{array}{l} S_{i}-P_{v1},\;0\;<\;S_{i}\;\leq \;8\\ S_{i}-P_{v2},\;8\;<\;S_{i}\;\leq \;10 \end{array}\right.$$

As for the punishment mechanism, *P_v_* is the adjustment parameter of the punishment value. By setting $P_{v1}\;<\;P_{v2}$, malicious attackers can be prevented from attacking the candidate node’s credit shell many times. As for the failure of the master node in the consensus process, the credit value will be directly deducted by 5 points, and the failed master node will be removed from the candidate queue and become a common node so as to avoid selecting the failed node for many times in a long time. When the same node assumes the master node for multiple rounds, it is necessary to reset the credit value of the node and assign the interval $0\;<\;S_{i}\;\leq \;8$ to avoid the problem of excessive centralization of the system.

By dynamically adjusting rewards value, penalties value *T_v_* and *P_v_*, the candidate queue can be regulated to avoid having too many or too few candidate queues. The candidate node queue gives the honest node with a higher credit value the chance to act as the master node first, which greatly reduces the possibility of the failed node rotating as the master node. The master node is selected from the candidate queue with a higher credit value, thereby greatly reducing the number of view switching. In order to ensure the normal operation of the consensus process and to avoid the possibility of no replaceable candidate nodes when the master node fails, the sorting of the candidate queue and the consensus process need to be performed synchronously.

7. Consistency protocol simplification.

The traditional PBFT consensus mechanism requires two communications with *O*(*N*^2^) complexity within the time to complete a round of consensus. There are many nodes in the grain food supply chain. If the master node is a Byzantine node, the communication overhead of the re-election of the master node will greatly affect the consensus efficiency. Therefore, while introducing the node credit evaluation mechanism, the consistency protocol of the CPBFT mechanism is simplified, which effectively reduces the communication complexity among nodes to *O*(*N*) level and further improves consensus efficiency to be suitable for application scenarios of grain food supply chain with many nodes.

**Figure 6:**
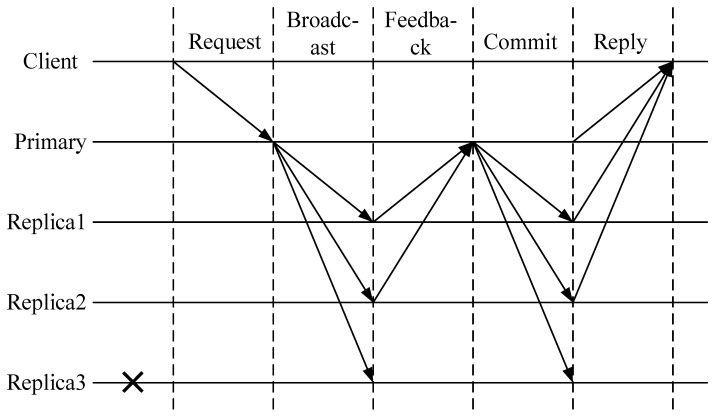
Simplified conformance protocol.

The simplified consistency protocol is shown in Figure 6. The specific steps are as follows.

- Step 1: The master node is selected through the node credit evaluation mechanism, and the master node presides over the current round of views, which is mainly responsible for message verification and the generation of new blocks after reaching a consensus.
- Step 2: The client sends request $<request,\;o_{c},\;t_{c},\;c_{c}>$ to the master node. *o_c_* is the request state executive machine, and *t_c_* is the time cutoff, and *c_c_* is the client number.
- Step 3: The master node broadcasts $<broadcast,\;v_{c},\;n_{c},\;d_{c},\;>m_{c},\;s_{c}>$ to the whole network. *v_c_* is the view number, *n_c_* is the message number, *d_c_* is the message digest, *m_c_* is the client request information, and *s_c_* is the credit value of each node. If each slave node approves the content of the certificate, it will reply to the approval information $<feedback,\;a_{ci}>$ to the master node, *a_ci_* is the approval information with the node number *i*.
- Step 4: If the master node receives no less than *2f* pieces of approval information, it will package the approval message <feedback, a> and send it to each slave node, where *a_c_* is the packaged approval information of each node. The slave node verifies whether the approval information of other slave nodes is correct and gets confirmed after passing the verification.
- Step 5: When the client receives at least *2f* + 1 pieces of confirmation information, it means a consensus has been reached, and the new block created by the master node is linked to the blockchain.

The simplified consensus protocol is combined with the node credit evaluation mechanism to encourage nodes to participate in the consensus process honestly, effectively reducing the possibility of a faulty node becoming the master node. At the same time, the communication complexity is reduced at the communication level of each node, and the consensus efficiency of the system can be significantly improved from two aspects. The optimized CPBFT algorithm is more suitable for complex scenarios in the grain food industry.

#### 3.3.2. Inter-Chain Consensus Based on Zero-Knowledge Proof

To ensure the privacy of cross-chain data, in this section, the Groth16 protocol is used to construct a zero-knowledge proof consensus protocol scheme. The communication complexity of the protocol is a constant number of group elements, and the verification complexity can also be reduced to a constant number of pairing operations, which meets the needs of high throughput and low storage of the blockchain.

As a bridge of communication among various business chains, the relay chain completes the transmission of private data ciphertext through DRSC and PESC and completes the exchange of shared information between enterprises. Although this process is carried out in the form of ciphertext, the key is transmitted together with the ciphertext. Once the key is leaked, the shared information is at risk of being tampered with. Although class III privacy information belongs to the shared information between business chains, it may cause serious losses if it falls into the hands of malicious attackers. Therefore, a consensus mechanism ZKP combined with zero-knowledge proof protocol is designed, it is assumed that the protocol is not falsifiable and the required parameters are generated by a trusted third-party module. While reaching a consensus between enterprise chains, the consensus does not reveal any relevant information about private data. Compared with the traditional Bitcoin blockchain based on PoW, the consensus is no longer achieved through expensive computing power, but the verifier verifies the proof generated by the prover, which is not only invisible to private data information but can also significantly reduce computing costs.

In the consensus process on the relay chain, two types of identity nodes are mainly divided, namely the prover node that shares the level III privacy information and the validator node that receives the level III privacy information. After judging the integrity of the business interaction data, the PESC sends the ciphertext and key to the business interaction initiation node corresponding to the relay chain, which is the prover node of the consensus process. The consensus process based on zero-knowledge proof on the relay chain mainly includes three stages, namely the trusted initialization stage Setup of system parameter setting, the generation proof stage GenProof of prover node *P*, and the verification proof stage VerProof of validator node *V*. The validator node that prioritizes the completion of evidence verification becomes the master node of the consensus, which is responsible for the packaging of this round of consensus transactions and the release of new blocks. In order to avoid the centralization of computing power, the validator node who has served as the master node for one round will be disqualified from issuing new blocks in the next three rounds of consensus, and other validator nodes that can verify the evidence π fastest will serve as the master node.

At the beginning of the consensus process, public input of the system needs to be introduced. Field F and arithmetic circuit $\mathbb{C}:F^{\left|x\right|+\left|w\right|}\to F^{\left|y\right|}$, in which *x* stands for the circuit input, *y* for the circuit output and *w* for proof. The public input $\left(x,\;y)=(c_{1},\;c_{2},\;\ldots ,\;c_{N}\right)$ and public output of |x|+|y|=N should be recorded. The prover node secret input: $w=(c_{N+1},\;c_{N+2},\;\ldots ,\;c_{m})$, record as set $I_{mid}=\{N+1,\;N+2,\;\ldots ,\;m\}$, in which $\left|w\right|=\left|I_{mid}\right|$. The three steps of the consensus mechanism are as follows. Before starting the zero-knowledge proof, the system-trusted initialization is performed.

8. Trusted initialization stage Setup
  (a) Construct the *QAP* string

The satisfiable problem of the arithmetic circuit ℂ is reduced to a *QAP* (quadratic arithmetic program) satisfiable problem by a trusted third party. The corresponding *QAP* string *(t(z), U, W, Y)* is constructed according to circuit ℂ, including three groups of polynomials $U=\{u_{i}(s)\}$, $W=\{w_{i}(s)\}$, $Y=\{y_{i}(s)\}$ and the Target polynomial t(z). The scale of *QAP* is *m,* and the degree is *d*.

  (b) Generate corresponding parameters

Corresponding parameters are generated by trusted third-party modules. $G_{1}$, $G_{2}$ are two factors in a bilinear mapping group, corresponding to the generators *g* and *h*, and the bilinear mapping group $G_{T}$. The bilinear mapping relation *e* is defined as $e:G_{1}\times G_{2}\to G_{T}$. ${\left[a\right]}_{1}$ is denoted as $g^{a}$, ${\left[b\right]}_{2}$ as $h^{b}$ and ${\left[c\right]}_{T}$ as $e{\left(g,h\right)}^{c}$. $\alpha ,\;\beta ,\;\gamma ,\;\delta ,\;s\overset{\$}{\leftarrow }F$ is selected in the area F randomly.

  (c) Generate the CRS

A trusted third-party module generates a common reference string $\sigma =({\left[\sigma_{1}\right]}_{1},\;{\left[\sigma_{2}\right]}_{2})$ and simulates a trapdoor τ=(α, β, γ, δ, s). ${\left[\sigma_{1}\right]}_{1}$ is an element on the elliptic curve $G_{1}$, and ${\left[\sigma_{2}\right]}_{2}$ is an element on the elliptic curve $G_{2}$. $\left(\sigma_{1},\;\sigma_{2}\right)$ is calculated by Equations (29) and (30).

(29) $$\sigma_{1}=\left(\begin{array}{c} \alpha ,\beta ,\delta ,{\left\{s^{i}\right\}}_{i=0}^{d-1},{\left\{\frac{\beta u_{i}(s)+\alpha w_{i}(s)+y_{i}(s)}{\gamma }\right\}}_{i=0}^{N}\\ {\left\{\frac{\beta u_{i}(s)+\alpha w_{i}(s)+y_{i}(s)}{\delta }\right\}}_{i=N+1}^{m},{\left\{\frac{s^{i}t(s)}{\delta }\right\}}_{i=0}^{d-2} \end{array}\right)$$

(30) $$\sigma_{2}=\left(\beta ,\gamma ,\delta ,{\left\{s^{i}\right\}}_{i=0}^{d-1}\right)$$

It should be noted that after calculating the common reference string *σ*, it is necessary to destroy the toxic wastes *σ*_1_ and *σ*_2_ to prevent malicious attackers from using them to generate false but valid proofs, which will affect the system security through perjury.

9. Generation proof stage GenProof of prover node *P*.

The prover node *P* selects $r_{1},r_{2}\overset{\$}{\leftarrow }F$ randomly and calculates the evidence π according to Equation (31). The calculation of (*A*, *B*, *C*) is shown in Equations (32)–(34). *c*_I_ is the data input involved in all the zero-knowledge consensus, including different levels of privacy data and public data digest from different chains. The multiply-add operation of large numbers (Multiexp) of the elliptic curve needs to be conducted when calculating *A*, *B*, and *C*. After the proof is generated, the prover node broadcasts the proof to each validator node for verification through the consensus protocol.

(31) $$\pi =({\left[A\right]}_{1},{\left[C\right]}_{1},{\left[B\right]}_{2})$$

(32) $$A=\alpha +\sum_{i=0}^{m}c_{i}u_{i}(s)+r_{1}\delta$$

(33) $$B=\beta +\sum_{i=0}^{m}c_{i}w_{i}(s)+r_{2}\delta$$

(34) $$C=\frac{\sum_{i=N+1}^{m}c_{i}(\beta u_{i}(s)+\alpha w_{i}(s)+y_{i}(s))+h(s)t(s)}{\delta }+Ar_{2}+Br_{1}-r_{1}r_{2}\delta$$

10. Verification proof stage VerProof of validator node *V*.

The validator node *V* checks the evidence π by Equation (35) and outputs bit *b*_0_ after verification. Only if Equation (35) is established, that is, the validator passes the verification of the evidence π, it outputs *b*_0_ = 1; otherwise, it outputs *b*_0_ = 0.

(35) $$e({\left[A\right]}_{1},{\left[B\right]}_{2})\overset{?}{=}e({\left[\alpha \right]}_{1},{\left[\beta \right]}_{2})e\left(\sum_{i=0}^{N}c_{i}{\left[\frac{\beta u_{i}(s)+\alpha w_{i}(s)+y_{i}(s)}{\gamma }\right]}_{1},{\left[\gamma \right]}_{2}\right)e({\left[C\right]}_{1},{\left[\delta \right]}_{2})$$

When the node outputs *b*_0_ = 1, it is considered that the validator node has successfully verified the evidence π broadcasted by the prover node, and each verifier node feeds back the verification output *b*_0_ to the consensus master node. When more than 51% of the validator nodes in the blockchain network have completed the proof of the zero-knowledge proof evidence π, namely, the master node has received more than 51% of the successful verification output *b*_0_ = 1, the system is deemed to have reached a consensus. The main validator node writes the level III privacy information ciphertext and key into a new block and broadcasts it to other validator nodes. At this point, the round of consensus is over.

#### 3.3.3. Cross-Chain Zero-Knowledge Proof Consensus Based on Kafka

**Figure 7:**
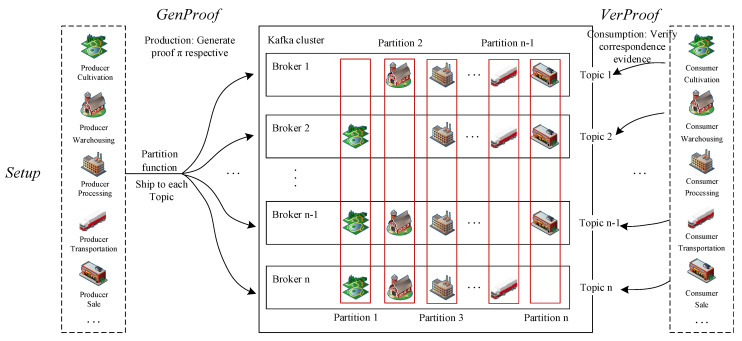
Consensus process of KZKP.

In the consensus mechanism based on a zero-knowledge proof of the consensus chain proposed in this study, each business chain needs to encrypt and transmit its business data to the consensus chain. The consensus chain completes the consensus on the business data of the supply chain process of the entire blockchain system. In the process of the zero-knowledge proof, each link node needs to verify the corresponding node evidence generated by all nodes except itself. The zero-knowledge proof consensus mechanism running on the relay chain is applied to a single prover scenario, and the scale of business data is relatively small. It is not suitable for the whole supply chain business data scenario in the consensus chain. Therefore, this section proposes the KZKP (Kafka-based Zero Knowledge Proof) consensus mechanism combined with the Kafka message queue and the zero-knowledge proof protocol. The partition mechanism of Kafka is used to effectively solve the complex verification problem of a single node for several points of evidence to realize the high parallel processing of the grain food supply chain complex data in the consensus process, which ensures data privacy and security while improving the efficiency of data consistency. The KZKP consensus process is shown in Figure 7.

The participants in the KZKP consensus process are the corresponding planting, warehousing, processing, transportation, and sales nodes on the consensus chain. Each node collects the business of the corresponding data representative chain through the PESC and the DRSC running on the relay chain, including the ciphertext, plaintext data digests and keys of privacy information at all levels. On the consensus chain, nodes participating in the consensus not only act as message producers to generate messages and send them to Topic, but also act as message consumers to pull messages from Topic.

Before the consensus process starts, it is still the trusted initialization process Setup of the system to generate the relevant parameters required for zero-knowledge proof. After the relevant parameters are generated, it enters the stage of evidence generation. Planting, warehousing, processing, transportation, and sales nodes are used as prover nodes to generate their own zero-knowledge proof evidence $\left(\pi_{1},\;\pi_{2},\;\ldots ,\;\pi_{k},\;\ldots ,\;\pi_{n}\right)$. Each node calls the producer API as a producer to send the specified Topic information, and the specified partition function sends the *n* − 1 evidences generated by each node to different broker agent nodes in a group. The partition function is defined in which each group of messages contains *n* − 1 of the n evidences, except itself. Broker1 receives $\left(\pi_{2},\;\ldots ,\;\pi_{n-1},\;\pi_{n}\right)$, Broker2 receives $\left(\pi_{1},\;\pi_{3},\;\ldots ,\;\pi_{n}\right)$, and so on, Broker *n* receives $\left(\pi_{1},\;\ldots ,\;\pi_{n-2},\;\pi_{n-1}\right)$. The cluster is vertically divided into *n* Partitions; each partition contains *n* − 1 copies of the proof evidence generated by the same node, which is distributed in different Brokers to achieve high parallel data processing for subsequent verification processes.

After the partition function partitions the evidence, it enters the stage VerProof. At this time, the identity of the planting, warehousing, processing, transportation and sales nodes on the consensus chain changes from message producers/provers to message consumers/verifiers. Each participating node constitutes a validator node consumer group, which pulls the corresponding evidence message from the corresponding Topic, and each link node verifies and feeds back the evidence corresponding to the private data generated by other link nodes except itself. The verifier node that first completes topic verification acts as the master node of this round of consensus to collect verification information of other partitions, and the master node will judge the output verification generated by each type of node. The master node also sets up an over-centralized protection mechanism to prevent the same node from serving as the master node for multiple rounds. When more than 51% of the evidence copies in each type of partition are successfully verified by the corresponding node, it is considered that the partition has achieved the consistency verification within the partition, and reports to the master node and broadcasts the proof to the nodes that have not been successfully verified. When the *n* partitions all reach the partition consistency, the consensus chain completes a round of consensus.

## 4. Results

### 4.1. Model Analysis

#### 4.1.1. Correctness Analysis

The research on the construction of grain food multi-chain blockchain based on zero-knowledge proof mainly includes two parts: the construction of a multi-chain model and the design of multi-chain collaborative consensus. Firstly, by studying the key information of each link of the whole grain food supply chain, this paper analyzes the access rights corresponding to various types of information and outputs a classification table of rights levels. The classification of privacy rights of information conforms to actual business scenarios. Secondly, a multi-chain blockchain network model for grain is constructed to isolate complex business relationships. One business chain corresponds to a type of related nodes with business associations and constitutes a multi-chain network together with the relay chain and consensus chain. Compared with the single-chain architecture of the blockchain, the multi-chain model effectively reduces the storage redundancy of the blockchain ledger and provides a feasible solution for the differentiated management and sharing of data.

In actual scenarios, not all of the data are suitable for public disclosure. Therefore, a hierarchical encryption and storage mode is designed for private data to implement hierarchical encryption for private data and public data to achieve differentiated data management and protection. The public data subject is stored in the off-chain database, which further reduces the burden of complex graphic data on the on-chain storage. The public data abstract is stored in the chain, forming a mapping relationship with the off-chain database, and the private data subject is stored in the corresponding chain through the data structure of the blockchain itself. In addition, the privacy and public data at all levels are analyzed. By comprehensively considering the privacy degree, the amount of data and the application scenarios, the privacy encryption method suitable for all levels of data is selected. A higher level of privacy data means a higher level of security, and privacy rights are backward-compatible, which effectively avoids the malicious behavior of non-relevant authority personnel on the privacy data. In addition, the relay cross-chain communication mode is designed as the medium of information transmission among chains, and the Schnorr signature is used to ensure the integrity of the data transmission process. When the data transmission is completed, the interactive data on the relay chain will be destroyed to avoid the risk of privacy data and key leakage.

Finally, the consensus steps of multi-chain collaboration are designed, including CPBFT consensus mechanism on the business chain, ZKP consensus mechanism on the relay chain, and KZKP consensus mechanism on the consensus chain. The consensus process is shown in Figure 4. Step 1 is the consensus process of the data in the business chain. Since the data in each link is complex and all need to participate in the consensus process, the CPBFT consensus mechanism is designed based on the credit evaluation, which greatly reduces the possibility of malicious nodes acting as master nodes. The consistency protocol is simplified to reduce the communication times of intra-chain nodes on the basis of retaining the original fault tolerance rate, thereby improving the consensus efficiency of complex data in the business chain. There is a specific data interaction between business chains and other chains; this step is carried out on the relay chain. In step 2, a consensus algorithm ZKP based on zero-knowledge proof is designed to achieve the consistency of interactive data without disclosing any relevant information about the data, thus ensuring the security of the private data involved in the interaction. Step 3 is the consensus step of the whole process data on the consensus chain. The zero-knowledge proof consensus mechanism KZKP based on the Kafka cluster is designed, which realizes the zero-knowledge proof of data between nodes in each link. The high parallel processing of the whole verification process is realized through the Kafka cluster, which improves the system consensus efficiency. So far, the multi-chain model has completed a round of information coordination consensus of the whole supply chain. The whole system model realizes the hierarchical protection and interaction of information data of the grain food supply chain, as well as the multi-chain information collaborative consensus of the whole process.

#### 4.1.2. Security Analysis

In the network layer, each business chain is naturally separated by the channel technology of fabric, forming an information barrier for each chain, which effectively ensures the difference and privacy of data information between each link. In the data layer, due to the numerous links in the grain food supply chain and complex business data, the business data is classified and encrypted by analyzing it. The privacy data of each authority level is protected by the cryptographic algorithm to ensure its security. The level I privacy information is encrypted by the AES encryption algorithm. The symmetric encryption algorithm is fast, but the data will be exposed directly once the key is leaked. Therefore, the ECC elliptic curve encryption algorithm is used to encrypt the AES symmetric key, and the security of the key is secured by the elliptic curve discrete logarithm problem. The level II privacy information is encrypted by Paillier homomorphic encryption algorithm. The algorithm has the characteristics of additive homomorphism, so it can directly operate the data ciphertext. The key security is based on the difficulty of large prime number decomposition.

The level III privacy information is encrypted by the EGamal homomorphic encryption algorithm, which also ensures the security of privacy data through the discrete logarithm problem. Regulatory information overlaps with privacy data at all levels, and the authority is relatively independent, so the RSA encryption algorithm is used, and the security of the algorithm is based on the decomposition of large prime numbers. The public information uses SHA256 to take the hash abstract and forms a mapping relationship with the off-chain database. The security is based on the unidirectionality and collision resistance capability of the hash function. In the process of data cross-chain interaction, the non-interactive Schnorr signature authentication protocol is adopted. This process is zero-knowledge and does not reveal any relevant information about the interactive data so as to ensure the security of the data interaction process.

In the consensus layer, the consensus of the business chain adopts the practical Byzantine fault-tolerant consensus CPBFT based on the credit evaluation. The credit value eliminates the potential of malicious nodes before the consensus, and the reward and punishment mechanism encourages consensus nodes to participate in the consensus process honestly, greatly reducing the node failure rate. In addition, the consistency protocol of CPBFT is simplified, and the fault tolerance rate of the consensus algorithm to Byzantine nodes is still 33%. The ZKP and KZKP consensus processes of the relay chain and the consensus chain are both implemented based on the unfalsifiable Groth16 protocol. The characteristics of the zero-knowledge proof make the certifier participating in the proof must master knowledge, and the verifier must be honest. The proof process will not disclose any relevant information of knowledge. For public parameters, it will be destroyed after generating corresponding system parameters so that illegal elements cannot generate false evidence through the parameters, thus ensuring the security of data interaction and information collaborative consensus.

#### 4.1.3. Scalability Analysis

The research on the construction of grain food multi-chain blockchain based on zero-knowledge proof shows that this research is not only applicable to various food scenarios but also applicable in the food field and supply chain system. The business chain and the nodes on the chain can be dynamically adjusted according to the actual situation. The data protection mechanism also conforms to the application scenario with high demand for business information privacy. The trusted cross-chain communication mode will ensure the integrity of the data transmission process. The CPBFT consensus based on credit evaluation can deal with scenarios with many business chain nodes. The consensus algorithms ZKP and KZKP based on zero-knowledge proof can also protect the privacy of data well. The Kafka cluster of KZKP consensus can dynamically add or delete participating nodes, and its message processing mechanism can effectively deal with complex proof concurrent verification problems.

### 4.2. Consensus Performance Analysis

**Figure 8:**
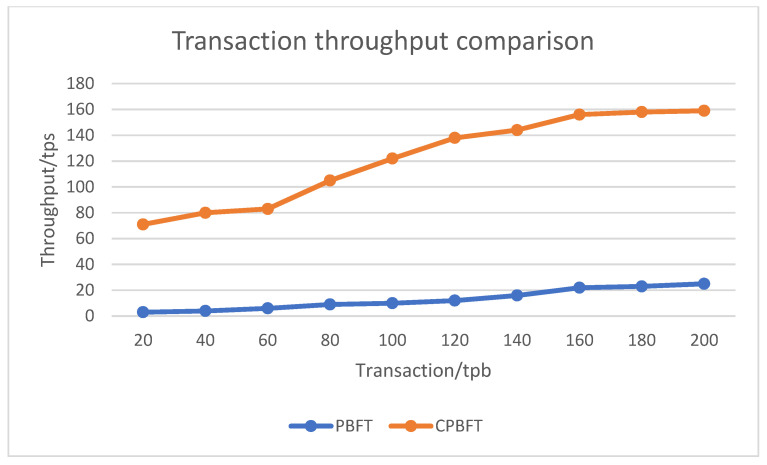
Comparison of CPBFT and PBFT transaction throughput.

This section analyzes the performance of the improved CPBFT and makes experimental comparisons mainly from the perspective of the throughput and transaction delay of CPBFT and PBFT algorithms. Five nodes are set up on the business chain to conduct a stress test to the consensus algorithm. The throughput as an evaluation index of transaction confirmation rate can intuitively reflect the ability of consensus to handle business, while the delay represents the timeliness of message sharing. As shown in Figure 8, the horizontal axis shows the number of transactions carried by a single block. With the increase in the number of transactions, the throughput of the two algorithms also rises. However, the improved algorithm in this paper has obvious advantages over the traditional PBFT in terms of throughput. When the number of transactions is 100 tpb, the throughput reaches 122 tps, and when the number of transactions is 200 tpb, the throughput reaches 159 tps, which is much higher than the PBFT consensus.

**Figure 9:**
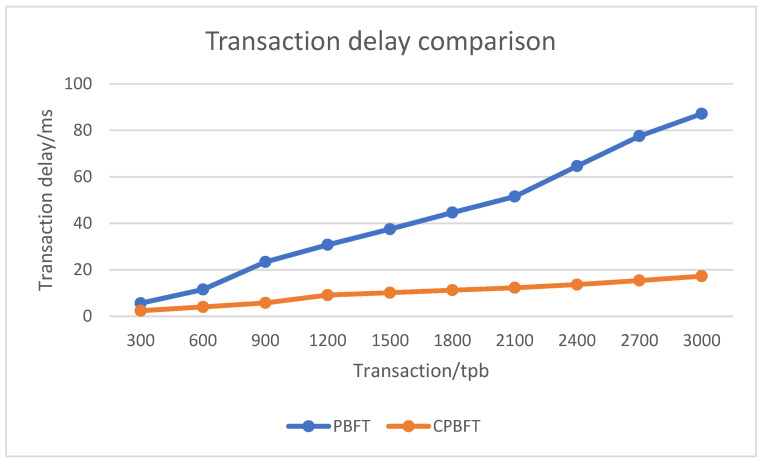
Comparison of CPBFT and PBFT transaction delay.

Furthermore, the transaction delay of the two algorithms is compared. As shown in Figure 9, the horizontal axis shows the number of transactions carried by a single block. As the number of transactions increases, the delay of the two algorithms also rises. However, the delay of the traditional PBFT consensus increases significantly, but that of the CPBFT of the simplified consensus protocol in this paper is not that obvious. When the transaction volume is 1500 tpb, the PBFT latency is 37.5 ms, and the CPBFT is only 10.1 ms. When the transaction volume is 3000 tpb, the CPBFT latency is 17.2 ms, and the PBFT has reached 87.4 ms. The consensus algorithm with a long delay will seriously affect the system’s performance. Compared with the PBFT algorithm, CPBFT has a shorter delay which can effectively cope with the grain food supply chain scenario with the increasing amount of data.

In addition, we made an analysis and comparison of the performance of PoW, PoS, PBFT, CPBFT, ZKP, and KZKP from four aspects—decentralization, scalability, security, and consensus efficiency, as shown in Table 2. The degree of decentralization is influenced by several factors—the number of participating nodes, the way the master node is selected and the weight of the consensus node. The higher the number of participating nodes, the more decentralized the power of nodes and the higher degree of the decentralization of the system. The weight of the consensus node refers to the probability that each node becomes the main node. Scalability includes two factors: resources consumption and communication complexity. Security includes the fault tolerance rate, attack diversity and attack cost. The fault tolerance rate refers to the proportion of malicious nodes that can withstand under the premise of reaching a consensus. Consensus efficiency contains two indicators: transaction delay and transaction throughput. The delay refers to the time it takes for a block to go from generation to consensus completion, and the higher the number of transactions processed per unit of time, the better the throughput performance of the system.

In terms of the decentralization, CPBFT evaluates node weights through a credit evaluation mechanism. High credit nodes have higher weights and nodes with high credit scores are not allowed to serve as master nodes for multiple rounds to avoid over-centralization. ZKP and KZKP consensus is based on the node that preferentially completes zero-knowledge proof as the master node and cannot serve as the master node for the next few rounds. Each node has the same weight. In regard to scalability, PoW and PoS consume too many resources, and the performance of a single node is limited, so they are not applicable to complex food security scenarios. The CPBFT communication complexity is reduced by simplifying the conformance protocol, and it inherits the low resource consumption characteristics of traditional PBFT. ZKP consensus on the relay chain enjoys moderate resource consumption and communication complexity. In the consensus chain, each type of node is involved in the process of verifying the evidence generated by other nodes, so they consume a large amount of computing resources. However, the partition feature of Kafka can effectively reduce the communication complexity among nodes. As for security, both CPBFT and PBFT can bear Byzantine malicious nodes of less than one-third of the total number of nodes. However, the credit evaluation mechanism effectively reduces the possibility of the existence of malicious nodes in the system. For ZKP and KZKP consensus, the public reference string (CRS) is destroyed after the corresponding parameter is generated, effectively avoiding the possibility of generating perjury, so the attack on the evidence is both costly and difficult. In a scenario where only three elements of the verification equation are proved, the integrity and reliability under the polynomial computing power must be guaranteed.

In terms of the aspect of consensus efficiency, since PoW and PoS need to compete for computing power and rights in the whole network, the lock-generating efficiency and throughput are low. CPBFT reduces the possibility of system failure, which enhances the consensus efficiency within the chain to some degree. The inter-chain ZKP consensus is mainly responsible for verifying the data interaction between two chains, which demonstrates less delay and moderate throughput. While the KZKP cross-chain consensus in the consensus chain can provide moderate transaction delay and throughput for the consensus with the high parallel processing characteristics of Kafka clusters.

### 4.3. Prototype System Verification

**Figure 10:**
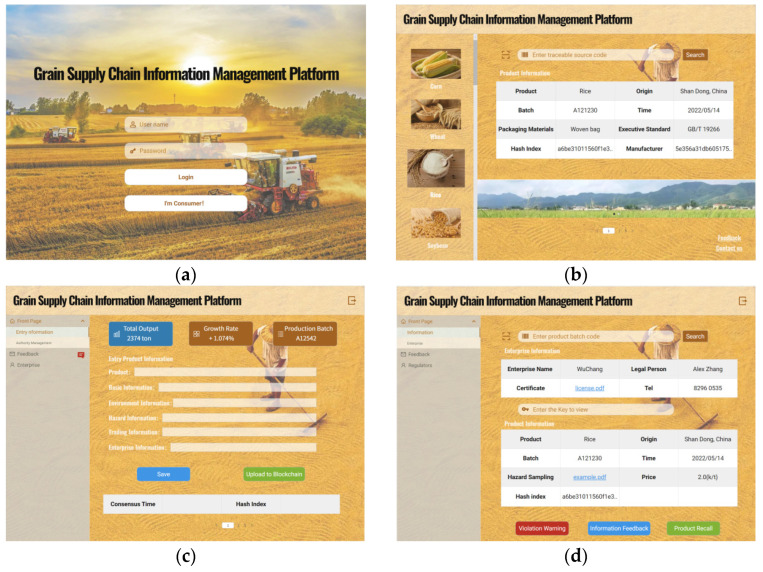
Schematic diagram of the prototype system interface. (**a**) Prototype system login interface; (**b**) consumer traceability interface; (**c**) enterprise information input interface; (**d**) regulatory interface for regulators.

The prototype system is implemented based on the Grain multi-chain blockchain network model in this paper, which adopts the development environment of Ubuntu version 20.04.1, Linux version 16.0.0, Docker version 20.10.7, Hyperledger Fabric 2.1 open-source framework, and Go language version 1.17.2. Four business chains are set up: the planting chain, including the planting link, the processing chain, including the purchasing, warehousing, processing, and packaging links, the storage and transportation chain, including the storage and transportation link, and the sales chain, including the sales link. In addition, it also involves a consensus chain responsible for all link information collaborative consensus and seven relay chains for cross-chain communication. Consumers can query traceability information, regulatory authorities can access regulatory information and supervise relevant data in real-time, and enterprises can use the prototype system to make permission access and encrypted interaction of private data. The specific implementation interface is shown in Figure 10.

Figure 10a shows the login interface of the prototype system. Enterprises or regulatory authorities log in through account passwords. Consumers do not need to log in. Click “I’m a Consumer!” to enter the consumer traceability function interface. Figure 10b shows the consumer traceability interface. Consumers can select the corresponding grain type, query the traceability information of products by scanning the code or entering the traceability code, and access the picture and video information in the production process at the bottom. Figure 10c is the information entry interface of participating companies, which includes five types of information and related information of the company. After filling in, the consensus step can be performed on the chain. At the bottom of the interface, the hash index and consensus duration information can be obtained. Figure 10d is the operation interface of the supervision department for supervision. According to the relevant grain batch, the corresponding enterprise information can be queried. After obtaining the ciphertext corresponding to the supervision information, enter the decryption key to obtain the plaintext of the information and can warn the enterprise of violations. At the same time, the information on hazardous substances exceeding the standard will be fed back, and the defective products can be recalled.

### 4.4. Work Deficiencies

The grain food supply chain has the characteristics of multiple participants, complex links and complicated business information. The multi-chain model can effectively isolate different participants and ease the burden of information storage in each link to some extent. However, the business data grows linearly over time, which still poses some pressure on each link. As the number of participants increases, the data concurrency decreases. Solutions to this problem still need to be explored in the future.

On the other hand, this paper aims to build a data information management method based on blockchain multi-chain and zero-knowledge proof in food scenarios. However, it is difficult to achieve this multi-chain network model and zero-knowledge proof multi-chain consensus in the complete fabric network at present. In the result analysis and verification, the prototype system and relevant theoretical methods need to be achieved in a simple way, but it turns out that the blockchain system model has some limitations through the streamlined feedback verification. Therefore, we fully verify and improve the support for the theoretical model through the theoretical analysis with experimental simulation. In the future, on the basis of implementing specific network models and consensus algorithms, the performance test of the proposed consensus algorithm and complete system implementation verification must be further strengthened.

## 5. Conclusions

In order to strengthen the information coordination of the grain supply chain, this paper constructs a multi-chain blockchain information management model for grain food based on zero-knowledge proof and realizes the multi-chain collaborative consensus of information in each link of the grain supply chain, as well as the cross-subject management of data. Considering the characteristics of numerous participation links and complex business data of the grain supply chain, a multi-chain network model is first designed to realize the data isolation of each business link. Secondly, the hierarchical encryption and storage mechanism of privacy data is designed by analyzing the key information of each link in the food supply chain, and the privacy of data transmission is guaranteed with the cryptographic algorithm, and therefore the secure storage of data information in the grain supply chain is realized. In addition, a grain food multi-chain and cross-chain communication model based on the relay chain is designed, and the trusted interaction of data is realized through Schnorr’s digital signature. Based on the multi-chain network model, the consensus steps suitable for multi-chain collaborative consensus are designed, which include three steps: CPBFT, ZKP and KZKP. The design of the consensus mechanism effectively reduces the probability of malicious nodes participating in the consensus, provides the concealment of the consensus process for private data, and realizes the coordinated consensus of information security under the multi-chain process of the grain food supply chain. Finally, the correctness, security, scalability, and consensus performance of the model are analyzed, and the prototype system is implemented so that enterprises, regulators and consumers can realize data management, supervision and traceability query. The analysis results show that although this study has some limitations in the construction of the food supply chain, this model provides an effective data management research idea for various types of food with various links and complex data in the scenario of the food supply chain.

The application of blockchain technology to the supply chain system has become a research hotspot in recent years, and the application of this technology to the grain food field provides a new feasible solution for smart agriculture. However, the grain food supply chain is complex, and the implementation of combining it with blockchain technology still presents some challenges. This paper provides an information collaboration scheme for multi-chain scenarios, but the research on the control of information sources is not involved in this paper, which also needs the support of relevant laws and policies issued by the government. Meanwhile, there are still some limitations in the implementation and verification of the proposed theoretical model in this study. In future research, we will put more emphasis on the implementation of the Fabric network model and the implementation research based on the zero-knowledge proof consensus algorithm to give more practical support for the theoretical model proposed in this paper.

## Figures and Tables

**Table 1:** Research review table.

Category	Research Content	References
Research on agricultural products and food supply chains based on single-chain	Research on the challenges and opportunities of blockchain technology applied to agricultural products and food	[29,30,31,32]
	Mainly focuses on the application research of information traceability in the supply chain	[13,33,34,35,36]
	Mainly focuses on information control and supervision research in the supply chain	[15,37,38]
Research on agricultural products and food supply chain based on multi-chain	Mainly focuses on the research of multi-chain technology applied to agricultural products and food supply chain	[20,21,22,39,40]
Research on consensus algorithm of agricultural products and food supply chain based on blockchain	Mainly focuses on the optimization research of blockchain consensus algorithm applied to supply chain system	[11,27,28,41]
Application of a zero-knowledge proof in blockchain	Mainly focuses on the application research of zero-knowledge proof in blockchain information protection and verification	[42,43,44,45]

**Table 2:** Performance comparison of consensus algorithms.

Performance Index		PoW	PoS	PBFT	CPBFT	ZKP	KZKP
Decentralization	Number of Consensus Nodes	Whole Network	Whole Network	Whole Network	Whole Network	Whole Network	Whole Network
	Main Node Selection Method	Compete	Compete	Vote	Vote	Compete	Compete
	Consensus Node Weight	Unequal	Unequal	Equal	Unequal	Equal	Equal
Scalability	Resource Consumption	High	Medium	Low	Low	Medium	High
	Communication Complexity	O(N)	O(N)	O(N^2^)	O(N)	O(N)	O(N)
Safety	Fault Tolerance Rate	<50%	<50%	<1/3	<1/3	<50%	<50%
	Attack Diversity	High	High	Medium	Medium	High	High
	Attack Cost	High	High	Medium	Medium	High	High
Consensus Efficiency	Delay	High	High	Medium	Medium	Low	Medium
	Throughput	Low	Low	Medium	Medium	Medium	Medium

## Data Availability

The authors declare that the data supporting the findings of this study are available from the authors.

## Appendix A

**Table A1:** Key information and classification of each link in the grain food supply chain.

Links of Grain Food Supply Chain			Basic Information	Environmental Information	Hazard Information	Transaction Information
Cultivation			Grower info, food type, seed source, origin info, sow/harvest time, output info, harvest equipment info, pesticide/fertilizer information and usage records, operator info, raw grain batch/traceability code.	Soil info, ambient temperature/humidity, illumination intensity, rainfall, O_2_/CO_2_ concentration, solid waste, domestic and industrial wastewater, harmful gas.	Mycotoxins, pesticide residues, heavy metals, pests.	Seeds/fertilizer/pesticide price, total cost, raw grain sales price, staff cost.
Purchasing & Warehousing	Purchasing		Buyer info, purchase time, purchase quantity, hazard sampling info, operator info, purchasing batch/traceability code.	None.	Quality deterioration, mycotoxins, pesticide residues, heavy metals, pests.	Raw grain purchase price, warehousing cost/price.
	Warehousing		Warehousing time, inventory number, raw grain quality inspection info, product quantity, product category, fumigant information and usage records, release time, operator info, warehousing batch/traceability code.	Inside/outside temperature/humidity of the warehouse, grain food temperature/humidity, N_2_/O_2_/CO_2_/PH_3_ concentration.		
Processing			Processor info, processing time, equipment inspection record, processing specific info, raw grain quality inspection info, operator info, processing batch/traceability code.	Environmental temperature/humidity, O_2_/CO_2_/PH_3_ concentration.	Mycotoxins, pesticide residues, heavy metals.	Processing cost/price.
Storage		Packaging	Storage provider info, product source, product quality inspection info, packaging material info/certificate, packaging batch/traceability code.	Environmental temperature/humidity, O_2_/CO_2_ concentration, epidemic prevention info.	Quality deterioration, pest.	Packaging/storage cost, product sales price, staff cost.
		Storage	Put in/delivery of cargo from storage time, product quantity, product source, quality inspection number, operator info, storage batch/traceability code.			
Transportation			Logistics provider info, product quantity, vehicle info, route info, place of departure/arrival info, departure/arrival time, operator info, transport loss, transportation batch/traceability code.	Ambient temperature/humidity inside the carrier, O_2_/CO_2_ concentration inside the carrier, pollutant detection info.	Quality deterioration, pest.	Transportation cost/price, staff cost.
Sale			Retailer info, product name/quantity, incoming/outgoing time, product batch/traceability code.	Environmental temperature/humidity, epidemic prevention info.	None.	Product purchase/sales price.

**Table A2:** Grading of privacy authority for key information in the grain food supply chain.

Classification of Key Information	Lv.I Privacy info	Lv.II Privacy Info	Lv.III Privacy Info	Public Info	Regulatory Info
Basic information	Seed source, operator info, hazard sampling info, raw grain quality inspection info.	Origin info, harvest equipment info, pesticide/fertilizer/fumigant information and usage records, quantity info, purchase time/quantity, sow/harvest/warehousing/release incoming/outgoing time, inventory number, equipment inspection record, processing specific info, vehicle info, transport loss.	Grower/Buyer/Processor/Storage provider/Logistics provider/Retailer info, raw grain/purchasing/warehousing/processing/packaging/storage/transportation/product batch code, product source, quality inspection number, route info, place of departure/arrival info, departure/arrival time.	Food type, product category/name, raw grain/purchasing/warehousing/processing/packaging/storage/transportation/product traceability code, packaging material info/certificate.	Grower/Buyer/Processor/Storage provider/Logistics provider/Retailer info, raw grain/purchasing/warehousing/processing/packaging/storage/transportation/product traceability code.
Environmental information	Solid waste, domestic and industrial wastewater, harmful gas, pollutant detection info.	Soil info, ambient temperature/humidity, illumination intensity, rainfall, N_2_/O_2_/CO_2_/PH_3_ concentration, inside/outside temperature/humidity of the warehouse/grain food, epidemic prevention info, ambient temperature/humidity inside the carrier, O_2_/CO_2_ concentration inside the carrier.	None.	None.	None.
Hazard information	Quality deterioration, mycotoxins, pesticide residues, heavy metals, pests.	None.	None.	None.	Exceed standard hazards sampling info.
Transaction information	Cultivation/warehousing/processing/packaging/storage/transportation/staff cost	Seed/pesticide/fertilizer price, raw grain/product purchase price, processing/warehousing/transportation price.	Raw grain/grain sales price.	Product sales price.	Raw grain/grain/product sales price.

**Algorithm** **A1.** PESC
**Input**: *Data m* Judgment(*m*)//Privacy level judgment **If** Lv.1 privacy information Encrypt (*m*) with *AES&ECC* hybrid encryption algorithm/*AES&ECC* encryption for lv.1 info **If** Lv.2 privacy information Encrypt (*m*) with *Paillier* encryption algorithm//*Paillier* encryption for lv.2 info **If** Lv.3 privacy information Encrypt (*m*) with *ELGamal* encryption algorithm//*ELGamal* encryption for lv.3 info **If** Supervision information Encrypt (*m*) with *RSA* encryption algorithm//*RSA* encryption for supervision info **If** Public information Encrypt (*m*) with *Hash* algorithm//*Hash* algorithm for public info **Return** *Public key* to DRSC and *Private key* to Relay chain **Output** Ciphertext(*m*_I_/*m*_II_/*m*_III_/*m_s_*/*m_pub_*) to DRSC//Output ciphertext **Else** Error report

**Algorithm** **A2.** DRSC
**Input**: *Data m*; *Relay request*; *Sender ID*; *Receiver ID*; *Cross-chain batch*. **Receive** *Relay request* Judgment(*Sender ID*; *Receiver ID*)//Identity judgment **Return** *Cross-chain batch* Sign(*Data m*) by *Schnorr* signature//Data integrity verification Verify the signature **If** the *Data m* is intact **Call** PESC for encryption data plaintext//Privacy data encryption **Send** *Data ciphertext* to consensus chain//Inter-chain consensus process Inter-chain consensus process execution//Data consensus **Return** *Data ciphertext* to DRSC **Output** *Data ciphertext* and *key* to business chain or consensus chain//Data relay **Else** Error report//Data integrity loss
